# Internet and psychosocial interventions: What is the evidence?

**DOI:** 10.1192/j.eurpsy.2021.207

**Published:** 2021-08-13

**Authors:** H. Riper

**Affiliations:** Clinical, Neuro And Developmental Psychology, Vrije Universiteit, Amsterdam, Netherlands

## Abstract

**Abstract Body:**

Digital mental health, before, during and after the COVID-19 pandemic Over the last two decades the digital landscape of mental health care research and service innovation has gained momentum. This period is characterized by many successes’ stories but brilliant failures as well. Today, e-mental health is like a two-headed Janus. One side of his face illustrates the birth of innovative technologies that entered mental health service provision. In parallel, the evidence-base for the application of these new technologies, such as internet-based treatments for depression, has been established with effect sizes comparable to those of face-to-face treatments. The other side of his face shows, however, that eMental-health has not yet lived up to its’ full potential as its actual delivery, evaluation and implementation in routine care has proven to be a much longer and bumpier road than expected. The question addressed in this presentation will be ‘what does the future hold’? Acknowledging that futures are difficult to predict Heleen Riper nevertheless provides insights into how we may overcome some of these bumps by combining the best of two worlds, i.e. blending digital and face-to-face components into one integrated treatment approach. She will illustrate these new developments by virtue of the results of the H2020 European Comparative Effectiveness Study for Major Depression and current experiences with especially videoconferencing during the Covid-19 pandemic.

**Disclosure:**

No significant relationships.

